# Self-Efficacy and Motivation to Quit of Smokers Seeking to Quit: Quantitative Assessment of Smoking Cessation Mobile Apps

**DOI:** 10.2196/25030

**Published:** 2021-04-30

**Authors:** Nikita B Rajani, Nikolaos Mastellos, Filippos T Filippidis

**Affiliations:** 1 Department of Primary Care and Public Health Imperial College London London United Kingdom

**Keywords:** smoking cessation, mobile applications, self-efficacy, motivation to quit, mHealth, mobile phone

## Abstract

**Background:**

Decreasing trends in the number of individuals accessing face-to-face support are leaving a significant gap in the treatment options for smokers seeking to quit. Face-to-face behavioral support and other interventions attempt to target psychological factors such as the self-efficacy and motivation to quit of smokers, as these factors are associated with an increased likelihood of making quit attempts and successfully quitting. Although digital interventions, such as smoking cessation mobile apps, could provide a promising avenue to bridge the growing treatment gap, little is known about their impact on psychological factors that are vital for smoking cessation.

**Objective:**

This study aims to better understand the possible impact of smoking cessation mobile apps on important factors for successful cessation, such as self-efficacy and motivation to quit. Our aim is to assess the self-efficacy and motivation to quit levels of smokers before and after the use of smoking cessation mobile apps.

**Methods:**

Smokers seeking to quit were recruited to participate in a 4-week app-based study. After screening, eligible participants were asked to use a mobile app (Kwit or Quit Genius). The smoking self-efficacy questionnaire and the motivation to stop smoking scale were used to measure the self-efficacy and motivation to quit, respectively. Both were assessed at baseline (before app use), midstudy (2 weeks after app use), and end-study (4 weeks after app use). Paired sample two-tailed *t* tests were used to investigate whether differences in self-efficacy and motivation between study time points were statistically significant. Linear regression models investigated associations between change in self-efficacy and change in motivation to quit before and after app use with age, gender, and nicotine dependence.

**Results:**

A total of 116 participants completed the study, with the majority being male (71/116, 61.2%), employed (76/116, 65.6%), single (77/116, 66.4%), and highly educated (87/116, 75.0%). A large proportion of participants had a low to moderate dependence on nicotine (107/116, 92.2%). A statistically significant increase of 5.09 points (95% CI 1.83-8.34) from 37.38 points at baseline in self-efficacy was found at the end of the study. Statistically significant increases were also found for the subcomponents of self-efficacy (intrinsic and extrinsic self-efficacies). Similarly, a statistically significant increase of 0.38 points (95% CI 0.06-0.70) from 5.94 points at baseline in motivation to quit was found at the end of the study. Gender, age, and nicotine dependence were not statistically significantly associated with changes in self-efficacy and motivation to quit.

**Conclusions:**

The assessed mobile apps positively impacted the self-efficacy and motivation to quit of smokers making quit attempts. This has important implications on the possible future use of digitalized interventions and how they could influence important psychological factors for quitting such as self-efficacy and motivation. However, further research is needed to assess whether digital interventions can supplement or replace traditional forms of therapy.

## Introduction

Smoking is a significant risk factor for many health problems, including lung cancer, heart disease, stroke, and asthma [1]. Although a majority of smokers want to quit smoking in the United Kingdom, the number of smokers making quit attempts has fallen over the past 10 years [2]. Of those individuals trying to quit, the majority are doing so without assistance, although this is associated with lower success rates [3,4]. Among a variety of cessation methods, pharmacological treatment that targets the biological element of addiction, combined with behavioral support, has been found to be the most effective [5]. Some studies have shown that individuals who receive combined behavioral and pharmacological support are 3 times more likely to quit compared with individuals quitting unaided [6]. Behavioral support interventions, particularly individual and group counseling, often use techniques targeting self-efficacy and motivation to quit of smokers, which are factors found to increase the likelihood of smokers making quit attempts and successfully quitting [7-11].

In the context of smoking cessation, self-efficacy is defined as a smoker’s confidence in their ability to refrain from smoking when faced with internal (intrinsic self-efficacy) and external stimuli (extrinsic self-efficacy) [12]. Research has found that incorporating self-efficacy into behavioral interventions and integrating it as a central component to the quitting process is effective in treating tobacco use and nicotine dependence [13]. Similarly, enhancing motivation to quit has been found to be a vital part “of the overall treatment for tobacco addiction as it increases smokers’ enthusiasm, sense of purpose and will to quit” [14]. Motivation to quit considers the importance placed on quitting and the level of determination a smoker has to quit successfully at a given quit attempt [15].

Despite the effectiveness of face-to-face support targeting important psychological factors such as self-efficacy and motivation, not all individuals are willing or able to access face-to-face support. One study showed that the number of individuals accessing smoking cessation services provided by the National Health Service in the United Kingdom has been continuously declining in part because of budget cuts; a similar trend has been observed in other European countries [16,17]. Further research shows that the proportion of smokers who tried to quit and/or used behavioral support declined in 2017 compared with 2008 [2]. The reduction in the number of individuals accessing face-to-face support leaves a significant gap in the treatment options for smokers seeking to quit. Although some of the alternative cessation methods may also target self-efficacy and motivation to quit, these factors are particularly relevant to digital cessation support, which could be an alternative for face-to-face behavioral treatment.

The provision of digital solutions has gathered increased interest in the field of public health, concurrently with the declining use of behavioral support for smoking cessation. With increased ownership and use of smartphones, smoking cessation interventions delivered via smartphones could be a promising and cost-effective avenue to bridge this gap. One study even reported that in 2015, 400 smoking cessation mobile apps were available in the various app markets, and this number has most likely risen over the past years [18]. In general, research has found that mobile-based smoking cessation interventions, particularly interventions based on text messages, can positively impact smoking cessation outcomes [19]. Although the evidence for app-based interventions is not as robust, many studies have found positive effects of mobile apps on smoking cessation. For example, Ubhi et al [20] found that smokers who used a cessation app reported higher quitting rates than nonapp users. Despite the proliferation of available cessation apps and their potential to tackle the threat posed by the tobacco epidemic, there is still much to learn about their efficacy and impact on smokers seeking to quit.

Specifically, the literature on whether and how smoking cessation mobile apps can affect psychological success factors such as self-efficacy and motivation to quit is sparse. The few studies that had attempted to investigate this had relatively small sample sizes and/or were purely qualitative [21-23]. Consequently, to address the gap in the literature, this study aims to quantitatively investigate whether the use of smoking cessation mobile apps can positively impact the self-efficacy and motivation to quit of smokers seeking to quit, 2 vital factors for successful cessation.

## Methods

### Study Overview

Smokers seeking to quit were recruited to participate in a 4-week web-based study with no face-to-face contact. Study recruitment and data collection were conducted from June 2019 to July 2020. After an initial screening, eligible participants were asked to use 1 of 2 smoking cessation mobile apps, Kwit or Quit Genius. Participants were asked to complete questionnaires at 3 study time points: baseline (before using the app), midstudy (2 weeks after using the app), and end-study (4 weeks after using the app). A follow-up questionnaire was sent 8 weeks after using the app. Participants were incentivized to participate in the study by providing them with free access to smoking cessation apps with all premium features and the chance to win a £50 (US $68) Amazon voucher.

### Recruitment

Participants were recruited via social media and posters across public places in London. Interested participants were sent a screening questionnaire to assess their eligibility. Participants who were 18 years or older, proficient in English, current smokers (at least 100 cigarettes smoked in their lifetime and smoked at least one cigarette a day), trying and willing to quit, not using other forms of smoking cessation treatments (including mobile apps), not using or had never used the apps Quit Genius or Kwit, and were not diagnosed with a mental health condition were eligible to participate.

### Sample Size

The research presented in this paper is part of a broader study exploring the use of gamification in smoking cessation mobile apps. Therefore, the sample size was calculated based on a previous study that investigated the impact of gamification elements in a fitness mobile app [24]. With a power level of 1−*β*=.80 and a significance level of *α*=.05, approximately 112 participants were needed to detect the impact of gamification in the context of the broader study. After accounting for a 20% dropout rate, we aimed to recruit 140 participants and required at least 112 participants to complete the study. Participants completed the study if they self-reported to engage with the app at least once a week for all study weeks and completed all required questionnaires.

### Mobile Apps and App Assignment

Participants were asked to use 1 of the 2 smoking cessation mobile apps based on their assigned participant identification number (PID). PIDs were assigned on receipt of informed consent and completion of eligibility screening. Participants with even-numbered PIDs were assigned to the mobile app Quit Genius, and participants with odd-numbered PIDs were assigned to the mobile app Kwit. As the analysis presented in this paper is from a broader study investigating the impact of gamification, both apps were chosen based on their embedment of gamification features and adherence to cessation guidelines in the United Kingdom [25]. More specifically, the mobile apps were chosen based on a comprehensive review of smoking cessation mobile apps available on the UK Android and iOS market [25]. The review screened for popular smoking cessation apps based on a minimum rating (ie, rated at least four out of five) and a minimum number of ratings (ie, 5 individual ratings). After identifying popular apps on the market, the apps were evaluated for their functionalities, adherence to smoking cessation guidelines, and integration of gamification features. The review found that the majority of popular smoking cessation apps had low adherence to evidence-based guidelines. Kwit and Quit Genius were selected because of their high level of adherence to smoking cessation guidelines and adoption of gamification features (relevant for the broader study focusing on gamification).

#### Quit Genius

**Figure 1:**
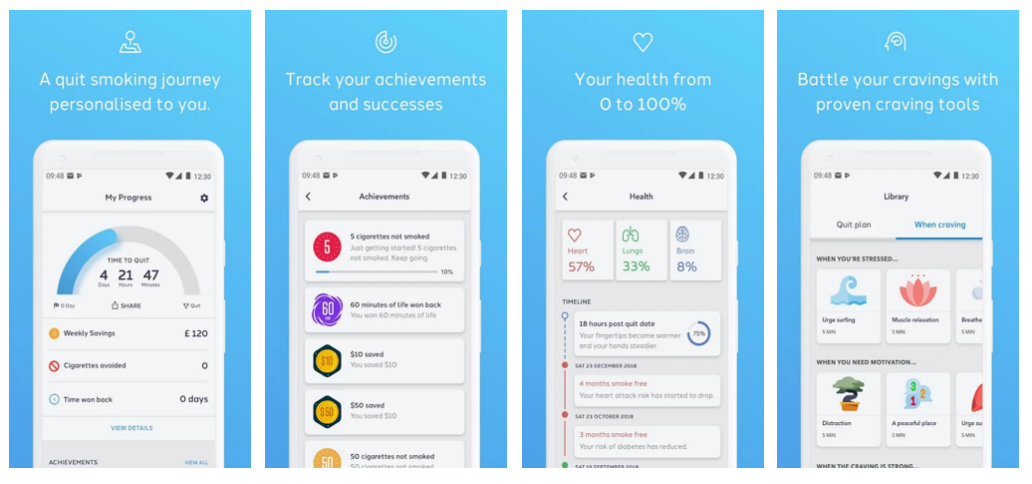
Screenshots of Quit Genius.

Quit Genius is a gamified smartphone mobile app targeted for smokers seeking to quit smoking and/or maintain their quit status [26]. It delivers personalized behavioral support to individuals based on the principles of cognitive behavioral therapy. The smoking cessation program includes videos, text, and audio recordings to help participants set goals and self-monitor. The app contains a calculator, tracker, cravings toolbox, cigarette diary to log cravings and triggers, and quit coach that provides personalized support. Participants from the study downloaded Quit Genius versions released from June 2019 (v.1.1) to July 2020 (v.1.9). Screenshots of the Quit Genius app are shown in Figure 1.

#### Kwit

**Figure 2:**
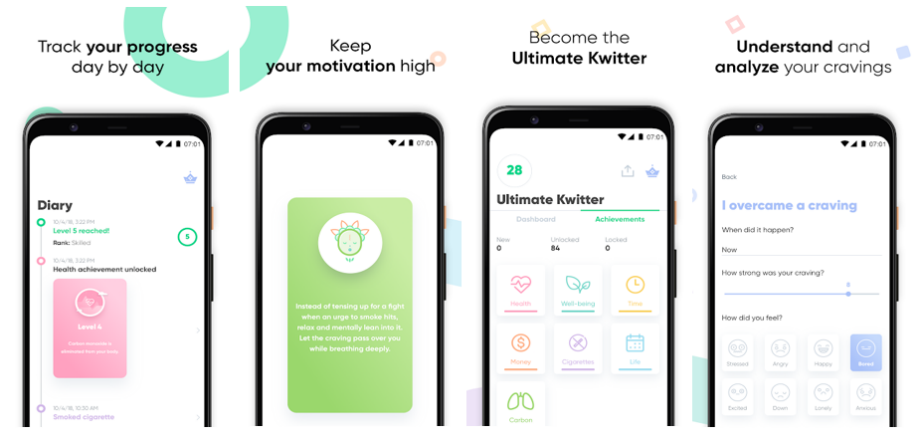
Screenshots of Kwit.

Kwit is a gamified and evidence-based smoking cessation mobile app that helps smokers quit and maintain their quit status [27]. The app is based on cognitive and behavioral therapy principles, gamification, and positive reinforcement. It includes a calculator tracker, motivation cards, social media sharing, and a smoking diary to log cravings and triggers. Kwit also contains features to help participants deal with relapses and self-monitor their journey to achieve their quitting goals. Participants from the study downloaded Kwit versions released from June 2019 (v.4.1) to July 2020 (v.4.4). Screenshots of the Kwit app are shown in Figure 2.

### Study Time Points

At baseline, participants were asked about general sociodemographic characteristics such as age, gender, marital status, and education. Participants were also asked about their current smoking habits, past quit attempts, self-efficacy, and motivation to quit. After app use, an assessment was conducted at 2 weeks (midstudy) and at 4 weeks (end-study). During both the midstudy and end-study assessments, participants were asked about their self-efficacy and motivation to quit. A follow-up questionnaire was sent at 8 weeks to assess app use, self-efficacy, and motivation to quit.

### Measures

#### Sociodemographic Factors

Several sociodemographic factors were assessed at baseline. These included age in years (18-29, 30-41, 42-53, or 54-65), gender (male or female), marital status (single, married, or civil partnered), residence categorized based on World Health Organization regions (Western Pacific, Americas, Southeast Asia, Europe, Africa, and Eastern Mediterranean) [28], education categorized based on the United Nations Educational, Scientific and Cultural Organization’s classification (low: primary school completed, medium: secondary school completed, and high: college or university degree) [29], and employment status (unemployed: individuals who are willing and able to work but have no employment; employed; nonemployed: individuals who are unable to work, including students and homemakers).

#### Nicotine Dependence

The 6-item Fagerström test was used to measure a participant’s tolerance and degree of dependence on nicotine [30]. Participants were classified into levels based on their nicotine dependency score: low (0-4 points), moderate (5-7 points), and high (8-10 points) [30,31].

#### Past Quit Attempts

Participants were asked how many serious attempts to stop smoking they made over the past 12 months. Participants were also asked if they used nicotine replacement products, prescribed medications, or mobile apps to help them quit during their past quit attempts in the previous year.

#### Self-Efficacy

The smoking self-efficacy questionnaire is a 12-item scale used to measure an individual’s confidence in their ability to refrain from smoking on a 5-point Likert scale with the following responses: not at all sure (1), not very sure (2), more or less sure (3), fairly sure (4), and absolutely sure (5) [32]. The total score ranged from 12 to 60, with higher scores indicating greater overall self-efficacy. The 2 subscales include intrinsic and extrinsic self-efficacy, with scores ranging from 6 to 30. Intrinsic self-efficacy refers to the confidence in one’s ability to refrain from smoking when faced with internal stimuli such as feeling depressed or anxiety, and extrinsic self-efficacy refers to the confidence in one’s ability to refrain from smoking when faced with external stimuli such as being with other smokers and drinking alcohol.

#### Motivation to Quit

Motivation to quit smoking is the level of importance a smoker places on quitting and the level of determination a smoker has to quit successfully at a given quit attempt [15]. Motivation to quit smoking was measured using a 2-item measure frequently used in past smoking cessation studies [33-35]. Participants were asked, “How important is it to you to give up smoking altogether at this attempt?” Responses included “desperately important,” “very important,” “quite important,” and “not all that important.” Participants were also asked, “How determined are you to give up smoking at this attempt?” Responses included “extremely determined,” “very determined,” “quite determined,” and “not all that determined.” Responses for both questions were coded and totaled, resulting in a range from 2 to 8, with a higher score indicating a higher level of overall motivation.

### Statistical Analysis

The analyses were conducted using STATA 13.1 (StataCorp). Descriptive statistics were used to present general participant characteristics, including current smoking habits and information on past quit attempts. Mean values of self-efficacy and motivation to quit were calculated at baseline, midstudy, and end-study. Owing to a low response rate of 40% to the follow-up questionnaire at 8 weeks, data from this questionnaire are not presented. Two-tailed paired sample *t* tests were used to test whether differences in self-efficacy and motivation to quit at various time points of the study were statistically significant. In addition, we explored various linear regression models to examine factors associated with changes in self-efficacy and motivation to quit. On the basis of an iterative process considering the fit of the model with the data, our linear regression models investigated the association between change in self-efficacy and change in motivation to quit before and after app use with age, gender, and nicotine dependence. Statistical significance was determined at 5% (*P*=.05), and 95% CIs were reported for all coefficients presented.

## Results

### Study Participants

**Figure 3:**
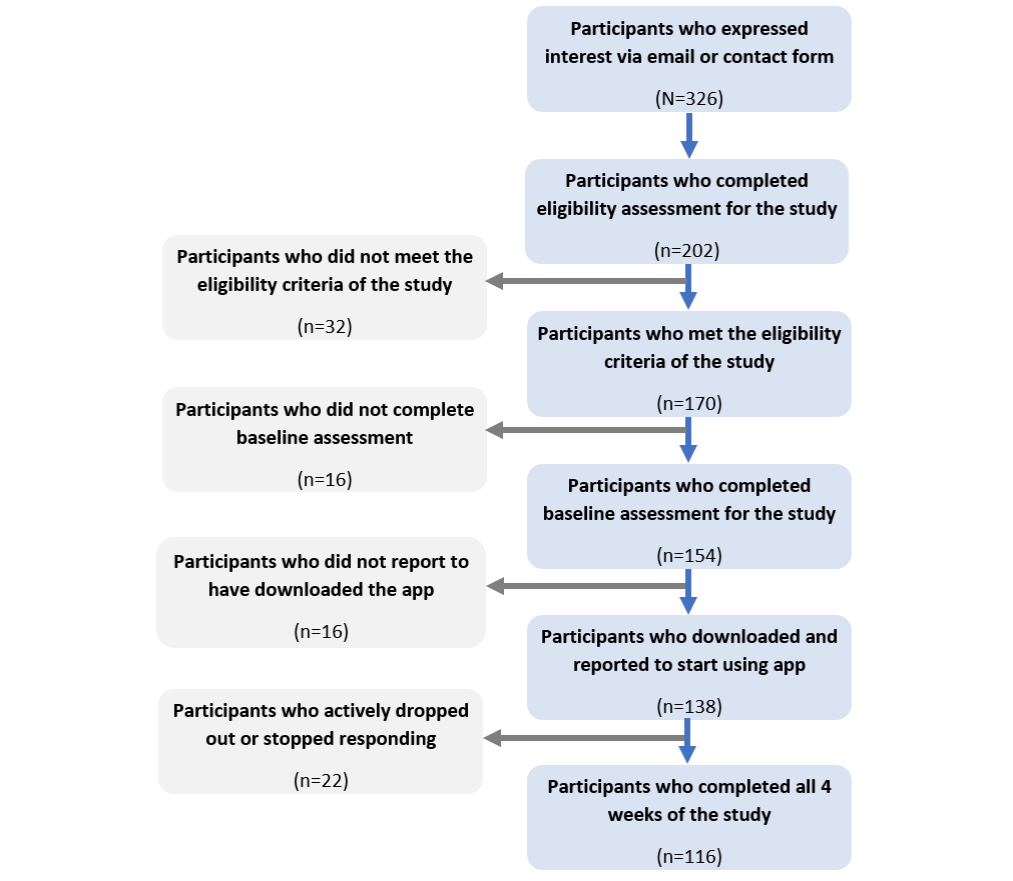
Overview of participant numbers from expression of interest to study completion.

The flowchart in Figure 3 displays the number of individuals completing the different stages of the study. Among the 326 individuals who expressed interest, 202 (62.0%) completed the eligibility questionnaire. Of the 202, 170 (84.2%) met the eligibility criteria. Of the 154 participants who completed the baseline assessment and were sent app installation instructions, 138 (89.6%) self-reported that they successfully installed and logged on to the assigned app. In total, 116 participants completed the entire study, and their characteristics are shown in Table 1.

**Table 1:** General characteristics, smoking habits, and past use of quitting aids (n=116).

General characteristics		Respondents, n (%)
**Age (years)**		
	18-29	49 (42.2)
	30-41	41 (35.3)
	42-53	15 (12.9)
	54-65	11 (9.5)
**Gender**		
	Male	71 (61.2)
	Female	45 (38.8)
**Education**		
	Low (primary school)	8 (6.9)
	Medium (secondary school)	21 (18.1)
	High (university or college degree)	87 (75.0)
**Marital status**		
	Single	77 (66.4)
	Married or civil partnered	39 (33.6)
**Employment status**		
	Employed	76 (65.5)
	Nonemployed	31 (26.7)
	Unemployed	6 (5.2)
	Prefer not to answer	3 (2.6)
**World Health Organization regions**		
	Western Pacific	4 (3.4)
	Americas	10 (8.6)
	Southeast Asia	16 (13.8)
	Europe	67 (57.8)
	Africa	17 (14.7)
	Eastern Mediterranean	2 (1.7)
**Daily smoking (number of cigarettes)**		
	≤10	63 (54.3)
	11-20	43 (37.1)
	21-30	8 (6.9)
	≥31	2 (1.7)
**Fagerström nicotine dependence**		
	Low (0-4)	62 (53.4)
	Moderate (5-7)	45 (38.8)
	High (8-10)	9 (7.8)
**Age (years) started smoking**		
	<15	30 (25.9)
	16-29	82 (70.7)
	≥30	4 (3.4)
**Past use of nicotine replacement therapy to quit**		
	No	64 (55.2)
	Yes	52 (44.8)
**Past use of cessation medication**		
	No	95 (81.9)
	Yes	21 (18.1)
**Past use of mobile apps to quit**		
	No	108 (93.1)
	Yes	8 (6.9)

Table 1 shows that the majority of participants were male (71/116, 61.2%), single (77/116, 66.4%), employed (76/116, 65.5%), and reported having a high level of education (87/116, 75.0%). With regard to smoking habits, more than half of the participants reported that they smoked 10 or fewer cigarettes daily (63/116, 54.3%). Similarly, the majority of respondents had low or moderate dependence on nicotine according to the Fagerström test (107/116, 92.2%) and reported that they did not use nicotine replacement treatments (64/116, 55.2%), medications (95/116, 81.9%), or mobile apps (108/116, 93.1%) in the past 12 months to aid with quit attempts.

### Self-Efficacy and Motivation to Quit

Table 2 displays the participants’ self-efficacy at baseline, midstudy, and end-study. After 4 weeks of using the app (end-study), intrinsic, extrinsic, and overall self-efficacy increased by 2.7, 2.4, and 5.1 points, respectively, compared with baseline values of 18.4, 19.0, and 37.4, respectively. Two-tailed paired sample *t* tests showed that these increases were statistically significant. Similarly, perceived importance, determination, and overall motivation to quite increased by 0.2, 0.2, and 0.4 points, respectively, compared with baseline values of 3, 2.9, and 5.9. These increases were statistically significant. However, the mean differences in self-efficacy and motivation to quit between midstudy and end-study were not statistically significant.

**Table 2:** Mean self-efficacy and motivation to quit scores across different study time points (n=116).

Characteristics			Baseline, mean (SD)		Midstudy, mean (SD)		End-study, mean (SD)		Baseline versus midstudy, mean difference (95% CI)		Baseline versus end-study, mean difference (95% CI)		Midstudy versus end-study, mean difference (95% CI)
**Self-efficacy**													
	Intrinsic (6-30)	18.4 (7.2)		20.6 (5.6)		21.0 (6.1)		2.2 (0.8 to 3.7)		2.7 (1.0 to 4.4)		0.5 (−0.7 to 1.6)	
	Extrinsic (6-30)	19.0 (7.0)		20.8 (6.2)		21.4 (6.3)		1.8 (0.2 to 3.4)		2.4 (0.7 to 4.1)		0.6 (−0.6 to 1.8)	
	Overall (12-60)	37.4 (13.3)		41.4 (10.5)		42.5 (11.5)		4.0 (1.2 to 6.8)		5.1 (1.8 to 8.3)		1.1 (−1.0 to 3.2)	
**Motivation**													
	Importance (1-4)	3.0 (0.8)		3.2 (0.8)		3.2 (0.8)		0.2 (0.0 to 0.3)		0.2 (0.0 to 0.4)		0.1 (−0.1 to 0.2)	
	Determination (1-4)	2.9 (0.8)		3.0 (0.9)		3.1 (0.9)		0.1 (−0.1 to 0.3)		0.2 (0.0 to 0.4)		0.1 (−0.1 to 0.2)	
	Overall (2-8)	5.9 (1.4)		6.2 (1.5)		6.3 (1.7)		0.2 (0.0 to 0.5)		0.4 (0.1 to 0.7)		0.1 (−0.1 to 0.3)	

The results of the linear regression models are shown in Table 3. Our analysis found that age was not statistically associated with a change in self-efficacy between end-study and baseline (*β*=−.07, 95% CI −.39 to .24). We also found that female participants self-reported a smaller change in self-efficacy compared with males; however, this result was also not statistically significant (*β*=−.91, 95% CI −8.09 to 6.26). Moreover, we found no association between a participant’s level of nicotine dependence and the change in overall self-efficacy. Similar to self-efficacy, our analysis found no statistically significant associations between age, gender, and nicotine dependence with change in overall motivation to quit between baseline and end-study.

**Table 3:** Linear regression models examining factors associated with change in self-efficacy and change in motivation to quit between end-study and baseline (n=116).

Variables			Change in overall self-efficacy (end-study vs baseline), *β* coefficient (95% CI)		Change in overall motivation (end-study vs baseline), *β* coefficient (95% CI)
Age (years)			−.07 (−.39 to .24)		−.02 (−.05 to .01)
**Gender**					
	Male	Reference		Reference	
	Female	−.91 (−8.08 to 6.26)		.17 (−.54 to .87)	
**Nicotine dependence**					
	Low	Reference		Reference	
	Moderate	−.01 (−7.07 to 7.04)		−.11 (−.81 to .58)	
	High	1.87 (−10.99 to 14.74)		.13 (−1.14 to 1.40)	
Constant			7.82 (−3.33 to 18.97)		.96 (−.14 to 2.06)

## Discussion

### Principal Findings

We found that self-reported self-efficacy and motivation to quit among participants using the assigned apps increased after app use. However, this increase largely occurred during the first 2 weeks of app use and then plateaued. We also found that age, gender, and nicotine dependence were not associated with changes in overall self-efficacy and motivation to quit between end-study and baseline.

The observed statistically significant increase in overall self-efficacy from baseline to midstudy and baseline to end-study implies that smokers seeking to quit smoking experienced an increase in perceived confidence in their ability to refrain from smoking. The same was found when examining intrinsic and extrinsic self-efficacy, suggesting that participants experienced increased confidence in their ability to refrain from smoking when faced not only with internal stimuli such as their feelings and cravings but also with external stimuli such as socializing with other smokers and drinking alcohol. This finding is important for smokers seeking to quit, as several studies have shown that high self-efficacy is associated with better smoking cessation outcomes [7-10].

The increase in self-efficacy after app use was generally consistent with previous studies. For example, one study examining the impact of a mobile app promoting smoking cessation in hospitalized patients found positive changes in self-efficacy among patients after app use [23]. However, pre- and post self-efficacy scores were only 45 minutes apart. Similarly, Hoeppner et al [21] found that among 30 participants, higher self-efficacy was reported 2 weeks after using a smoking cessation mobile app compared with baseline.

We also noted that the majority of the increase in self-efficacy was found between baseline and midstudy, after which self-efficacy levels stabilized. This could mean that the mobile apps may have a saturated effect by the midstudy point; therefore, the first 2 weeks act as a *ramp-up* phase, after which self-efficacy plateaus. Boardman et al [36] conducted a study among 600 African American smokers and found that the smokers continued to sustain high levels of self-efficacy and motivation scores up to 6 months post intervention. Although our findings are consistent with the findings from the study by Boardman et al [36], this study did not investigate the long-term maintenance of self-efficacy levels after mobile app use; therefore, this could be explored in future research.

Similar to self-efficacy, a statistically significant difference in determination, importance, and overall motivation to quit was found between baseline and end-study. This shows that participants experienced an increase in their perceived determination to quit smoking and how important they felt it was to quit at this attempt after using the app compared with baseline. Both apps contained several features that could have led to increased motivation. For example, possible features embedded in the Kwit app that could have led to higher motivation include unlocking achievements, motivation cards, trackers, and craving management tools. On the other hand, Quit Genius includes features such as achievement badges, cravings management, and a quit coach. Comparing the 2 apps and/or understanding which app-specific features were associated with changes in self-efficacy and motivation to quit was beyond the scope of our analysis.

The statistically significant increase in motivation to quit found in our analysis is in line with another study investigating the impact of Quit Genius, which also reported that participants had higher motivation to quit after using the app [22]. However, that study was purely qualitative, with only 15 participants using the app for 1 week. Similarly, Hoeppner et al [21] found increased motivation to quit levels among smokers prescribed to use a cessation app for 3 weeks. It is interesting to note that, on average, participants who enrolled in the study had relatively high motivation to quit at baseline, which could be because of participants with high motivation self-selecting into the study [37].

Furthermore, we did not find a statistically significant association between age, gender, and nicotine dependence with change in overall self-efficacy and motivation to quit. This suggests that the mobile apps had a similar effect on participants’ self-efficacy and motivation to quit at the end of the study compared with baseline, regardless of age, gender, and level of nicotine dependence. However, this might not be generalizable, as our sample had a majority of male, educated, and employed participants with low to moderate nicotine dependence. It may also have been underpowered to detect nuanced differences in effectiveness. A more diverse sample could allow for stronger inference on benefits of these apps across users of all demographics.

The positive impact of the mobile apps on the self-efficacy and motivation to quit of smokers highlights the importance and role of these psychological factors during a quit attempt. According to Elshatarat et al [13], self-efficacy is “a central concept of the quitting process” and is vital for achieving cessation and preventing relapse. Self-efficacy beliefs can determine how and when a smoker will initiate their coping behaviors and how long they are sustained when experiencing cravings or withdrawal symptoms [13]. Similarly, theories surrounding motivation to quit suggest that it is also a critical ingredient in quitting and plays an important role in the quitting process by enhancing a smoker’s intention to quit [11]. Studies have also found that apps can influence other psychological factors, such as well-being and psychological empowerment, to help smokers quit. For example, Lin et al [38] investigated the impact of Quit Genius on smokers seeking to quit and found that the app was able to enhance hedonic well-being and psychologically empower smokers, which, in turn, significantly increased the odds of successfully quitting.

Future research should continue to build upon our understanding of how mobile app solutions can positively impact psychological factors, such as self-efficacy, motivation to quit, and empowerment, which are found to be vital for successful cessation. For example, certain features or design elements may be more effective than others in influencing important psychological factors, promoting health behavior change, and improving quit rates. App developers and tobacco cessation and behavior change specialists could benefit from working together to develop effective digital cessation programs that contain features targeted at improving and enhancing psychological factors that may play a role in the quitting process. Finally, as past studies have shown that internet-based interventions can help the disadvantaged more, the possibility of providing effective digitalized interventions could help reduce health inequalities [39,40]. Consequently, more research that rigorously assesses the use and impact of digitalized behavioral interventions could be very valuable for public health policy makers working to attenuate health disparities.

### Limitations

One of the limitations of our study is that the majority of participants had low dependence on nicotine, which could affect baseline self-efficacy and/or motivation to quit. Future research could replicate the research on high-dependency smokers to see whether the results are generalizable. Similarly, unlike our study, future studies could also include participants with mental health conditions to ensure that the findings are generalizable to this population subgroup as well. Another limitation is the reliance on only self-reported data, which can lead to biases such as social desirability bias and, therefore, may not always be the most reliable. Moreover, our study had some methodological limitations. For example, participants were assigned to 1 of 2 mobile apps to ensure accurate data collection before and after app use. However, in reality, smokers would naturally self-select interventions on their app stores. It could also be that our study consisted of individuals with higher motivation than the general population, causing some volunteer bias. Finally, not enough follow-up data were collected because of low response rates at 8 weeks to be able to comment on long-term impact. Therefore, future studies could investigate whether the effects of mobile apps are sustained in the long term and how this can be compared with face-to-face behavioral cessation programs. Despite these limitations, this study develops a better understanding of the impact of smoking cessation apps and could provide a basis for future randomized controlled trials.

### Conclusions

In conclusion, we found that smoking cessation mobile apps could have a positive impact on important psychological factors associated with better cessation outcomes. This has important implications for the development and use of mobile apps as evidence-based support for smoking cessation. Although this research might provide insights for the development of future apps, further research is required to enhance our understanding of how digitalized interventions could positively impact self-efficacy and other psychological factors vital for successful cessation. The limitations of our study methodology highlight the issues that future research can address differently. More rigorous and evidence-based research is vital to determine whether digital interventions can supplement or replace traditional forms of therapy.

## Abbreviations

PID: participant identification number
